# Improving Healthy Daily Activity in Older Adults with Multimorbidity: Efficacy and Acceptability of ActiveOT

**DOI:** 10.1093/geroni/igaf122.524

**Published:** 2025-12-31

**Authors:** Tara Klinedinst, Juleen Rodakowski, Lee Jennings, Nicholas Hollman, Sarah Beth Bell, Zachary Pope, Zsolt Nagykaldi, Darla Kendzor

**Affiliations:** The University of Oklahoma Health Sciences Center, Oklahoma City, Oklahoma, United States; University of Pittsburgh, Pittsburgh, Pennsylvania, United States; University of Oklahoma Health Sciences, Oklahoma City, Oklahoma, United States; The University of Oklahoma School of Community Medicine, Tulsa, Oklahoma, United States; The University of Oklahoma Health Sciences Center, Oklahoma City, Oklahoma, United States; The University of Oklahoma Health Sciences Center, Oklahoma City, Oklahoma, United States; The University of Oklahoma Health Sciences Center, Oklahoma City, Oklahoma, United States; The University of Oklahoma Health Sciences Center, Oklahoma City, Oklahoma, United States

## Abstract

Nearly half of U.S. older adults have multimorbidity (≥2 chronic conditions) in addition to functional limitations that restrict engagement in health-promoting daily activity (e.g., sleep, medication-taking, physical activity). We tested a combination of behavioral activation and occupational therapy (n = 21) compared to brief education (n = 20) on performance of health-promoting daily activity, measured by the Canadian Occupational Performance Measure (COPM) at baseline, 10 weeks, and 22 weeks. “ActiveOT” was delivered by occupational therapists in 10 once-weekly sessions in participant homes. On average, the 41 participants were 73 years old, female, and identified as White. They averaged 4.0 chronic conditions (SD = 1.3), and 1.6 functional limitations (SD = 1.5). Baseline scores were not different between groups. At 10-weeks, the ActiveOT group had higher COPM scores (M = 7.3, SD = 1.7) compared to controls (M = 4.9, SD = 1.8; t(35)= 4.32, p < 0.001). This trend continued at the 22-week follow-up where the ActiveOT group had higher COPM scores (M = 7.8, SD = 1.2) than controls (M = 5.3, SD = 1.8; t (33) =4.81, p < 0.001). Between-group COPM scores reflected large effect sizes at 10 weeks (Cohen’s d = 1.42) and 22 weeks (Cohen’s d = 1.63), and 89% of ActiveOT participants experienced clinically meaningful improvements (MCID=3). We conducted semi-structured follow-up interviews with ActiveOT participants (n = 15) to understand the acceptability and satisfaction with the intervention and study processes. ActiveOT participants reported overall satisfaction with the intervention and appreciated the flexibility of goal areas and person-centered focus. ActiveOT participants demonstrated improved performance of health-promoting daily activity and evaluated the intervention favorably. Future work will focus on implementation of ActiveOT in primary care contexts.

